# 

**Published:** 1852-05

**Authors:** 


### WESTERN JOURNAL

OF

### MEDICINE AND SURGERY.

EDITORS.

---

LUNSFORD P. YANDELL, M. D.

PROFESSOR OF PHYSIOLOGY AND PATHOLOGICAL ANATOMY
IN THE UNIVERSITY OF LOUISVILLE.

AND

THEODORE S. BELL, M.D.

### RECEIPTS OF MEDICAL JOURNAL FOR APRIL, 1852.

| | |
|---|---|
| Dr. J. E. Shipp, Centerville, Tenn,, - - - | $5 00 |
| W. Killebrew Martin, Texas, - - - - - | 5 00 |
| B. Spaulding, Lebanon, Ky. - - - - - | 5 00 |
| Stoddard, Newburg, Ind., - - - - - | 5 00 |
| Drs. Kimbal & Meaners, Fort Henderson, Ala., - - - | 10 00 |
| Library Association, Cincinnati, Ohio, - - - - | 5 00 |

The WESTERN JOURNAL OF MEDICINE and SURGERY is published monthly by the undersigned, at the corner of Main and Fifth streets, Louisville, at $5 per annum, payable in advance. Each number contains 96 pages, making two volumes in the year of more than 550 pages each.

Contributions to its pages by the Physicians of the Valley of the Mississippi addressed to the Editors, are respectfully solicited.

Letters on business, to be addressed, postage paid, to the publishers.

PRENTICE & HENDERSON.

